# Spatially Aware Acquisition‐Independent Deep Learning for IVIM MRI Parameter Estimation in Patients With Esophageal Cancer

**DOI:** 10.1002/mrm.70592

**Published:** 2026-09-14

**Authors:** Daan Kuppens, Roman S. Oort, Sebastiano Barbieri, Stella Mook, Gert J. Meijer, Oliver J. Gurney‐Champion

**Affiliations:** ^1^ Department of Radiology and Nuclear Medicine Amsterdam University Medical Center Amsterdam the Netherlands; ^2^ Imaging and Biomarkers, Cancer Center Amsterdam Amsterdam the Netherlands; ^3^ Queensland Digital Health Centre University of Queensland Brisbane Australia; ^4^ Centre for Big Data Research in Health UNSW Sydney Sydney Australia; ^5^ Department of Radiation Oncology University Medical Center Utrecht Utrecht the Netherlands

**Keywords:** deep learning, esophageal cancer, IVIM MRI, neural controlled differential equations, parameter estimation

## Abstract

**Purpose:**

Neural controlled differential equations (NCDEs) recently emerged as a robust deep learning approach for quantitative MRI parameter estimation. NCDEs offer flexibility to changes in acquisition protocols by modeling the signal evolution dynamics. However, NCDE implementations operate on a voxel‐by‐voxel basis and cannot exploit spatial information, limiting effectiveness. The purpose of this study is to develop and validate an acquisition‐independent and spatially aware neural network for intra‐voxel incoherent motion (IVIM) MRI parameter estimation.

**Methods:**

Spatially aware NCDEs (Spatial NCDEs) were evaluated in simulations across a range of acquisition protocols and signal‐to‐noise ratios. Performance was compared with least squares (LSQ) fitting, segmented fitting, voxel‐wise NCDEs, and spatially aware neural networks (UNet). In patients with esophageal cancer, discriminative ability for predicting response to neoadjuvant chemoradiotherapy was examined.

**Results:**

Spatial NCDEs achieved lower mean squared error (MSE) for estimating IVIM parameters than LSQ, segmented fitting, 1D NCDE, and UNet. At SNR 20, MSE was 81%, 85%, and 83% lower than LSQ, 85%, 89%, and 88% lower than segmented fitting 62%, 71%, and 52% lower than 1D NCDE and 55%, 67%, and 44% lower than UNet for D, $D^{*}$, and f, respectively. In patients with esophageal cancer, Spatial NCDE‐based parameter estimates showed improved, though not statistically significant, discriminative ability for predicting response to neoadjuvant chemoradiotherapy compared to LSQ.

**Conclusion:**

Spatial NCDEs provide a robust, accessible solution for high‐quality IVIM MRI parameter estimation, enabling broader adoption of deep learning‐based parameter estimation.

## 1 Introduction

Intra‐voxel incoherent motion (IVIM) MRI utilizes diffusion‐weighted imaging (DWI) to quantitatively assess tissue diffusion and perfusion properties [1]. The use of the IVIM‐derived diffusion coefficient (D) and pseudo‐diffusion signal fraction (f) has demonstrated potential for tumor diagnosis and grading, therapy monitoring and as prognostic markers of clinical outcome in a range of cancer types [2, 3, 4, 5, 6, 7, 8, 9, 10, 11]. In particular, in esophageal cancer, early treatment‐induced changes in the apparent diffusion coefficient are suggested to precede anatomical changes and may therefore support prediction of response to neoadjuvant chemoradiotherapy [12, 13].

Despite these promising applications, the clinical utility of IVIM MRI is limited by the robustness of parameter estimation methods. Conventionally, IVIM parameters are estimated using voxel‐wise least squares fitting (LSQ), which fits parameter values from DWI images acquired with varying diffusion weightings (*b*‐values) by minimizing the sum of squared differences between the measured DWI signal and the modeled signal. The parameter estimates obtained using LSQ are sensitive to noise and variations in acquisition protocol [14, 15, 16], potentially getting stuck in local minima or overinterpreting noise. As a result, the repeatability and accuracy of IVIM MRI are adversely affected, leading to mixed results in the literature on the effectiveness of IVIM parameters as a biomarker [17]. Improving the robustness of parameter estimation methods is essential for the broader clinical utility of IVIM MRI.

Attempts to address the inherent limitations of LSQ include voxel‐wise neural networks [18, 19], and spatially aware neural networks [20, 21]. Voxel‐wise deep learning‐based methods improve the accuracy of IVIM parameter estimation, provide faster computation of parameter maps, and reduce day‐to‐day variability in patient measurements while preserving sensitivity to treatment [19, 22]. Incorporating spatial awareness into neural network architectures can further enhance the accuracy of IVIM MRI parameter estimation [20, 21]. However, unlike LSQ and other conventional fitting methods, deep learning approaches are not typically flexible to changes in acquisition protocol. Since neural networks have to learn the relationship between the measured signal and IVIM parameters strictly from data, changes in the measured signal not originating from an underlying difference in physiology but from changes in diffusion weighting are hard to account for. As a result, neural networks must be specifically trained and validated for each unique acquisition protocol, which requires specialized expertise and large, protocol‐specific datasets. This limits the development of generalizable solutions and poses a barrier to more widespread adoption of deep learning‐based quantitative MRI (QMRI) parameter estimation.

To alleviate the acquisition‐dependence of neural networks, we showed that neural controlled differential equations (NCDEs) [23] can handle variations in acquisition protocol by learning to approximate the underlying differential equation describing the changes in signal over changes in diffusion weighting [22]. However, because these NCDEs operate on a voxel‐by‐voxel basis, they cannot take advantage of spatial information from neighboring voxels, which puts a limit on their overall effectiveness. To the best of our knowledge, no spatially aware, acquisition‐independent implementation exists for deep learning‐based QMRI parameter estimation.

We hypothesize that adding spatial awareness to NCDEs will allow them to retain their generalization properties, while improving performance in estimating IVIM MRI parameters. Hence, we implement and validate spatially aware NCDEs (Spatial NCDEs) for IVIM MRI parameter estimation and demonstrate their performance both in simulated data and in vivo in patients with esophageal cancer. This implementation is a step toward accessible and high‐quality IVIM MRI parameter estimation using deep learning.

## 2 Methods

All analyses were performed using Python (v3.9, Python Software Foundation) and PyTorch (v2.8.0, PyTorch Foundation).

### 2.1 IVIM Model

In IVIM MRI, diffusion‐weighted gradients are used to sensitize the MRI signal to incoherent motion, such as diffusion and microvascular perfusion. In this model, the signal intensity (s(b)) at a given diffusion‐weighting (*b*‐value) is determined by the diffusion coefficient (D), which relates to tissue cellularity and structural integrity; the pseudo‐diffusion coefficient ($D^{*}$), and the pseudo‐diffusion signal fraction (f), both of which are associated with tissue vascularization and perfusion, and the baseline signal intensity ($s_{0}$), by:

(1) $$s\left(b\right)=s_{0}\left(\left(1-f\right)\;\exp\left(-\mathrm{bD}\right)+f\;\exp\left(-bD^{*}\right)\right)$$

### 2.2 Neural Networks

#### 2.2.1 Proposed Model: Spatial NCDE

We developed a Spatial NCDE (Figure 1) that can estimate spatially structured IVIM MRI parameter maps, independent of the acquisition protocol (the configuration of *b*‐values at which the diffusion‐weighted MRI signal is measured). NCDEs learn a parameterized mapping from a signal defined over a physical domain (i.e., diffusion weighting) to an output representing relevant descriptors of the measured signal (i.e., IVIM MRI parameters). Spatial NCDEs extend upon this framework by performing structured estimation over additional spatial domains. Our Spatial NCDE consisted of two fully connected neural networks ($l_{ø}^{1}$ and $f_{ø}$) and a convolutional neural network ($l_{ø}^{2}$). Together, these networks learned a mapping from 2D DWI images ($\tilde{S}$) to 2D output parameter maps $\tilde{y}$. The input sequence contained spatially structured pairs of DWI signal intensities and the *b*‐values at which these signals were measured (${\tilde{S}}_{i}=\left[{\tilde{s}}_{i},b_{i}\right]$). The signal intensity curve as a function of diffusion weighting on a voxel‐wise basis defined the knots of a continuously differentiable cubic spline (X), which was used as a control signal to the controlled differential equation. $l_{ø}^{1}$ initialized the hidden state ($z_{t_{0}}$) using the first element of the input trajectory ($X_{0}$). The components $l_{ø}^{1}$ and $f_{ø}$ remained consistent with the previously described formulation [22, 23], $l_{ø}^{1}$ as a multi‐layer perceptron responsible for initializing the hidden state, $f_{ø}$ was a multi‐layer perceptron (MLP) governing the vector field dynamics (solved using *NeuralCDE* (https://github.com/patrick-kidger/NeuralCDE) and *torchcde* (https://github.com/patrick-kidger/torchcde, v0.2.5) packages). We modified the architecture of $l_{ø}^{2}$ from an MLP into a UNet [24], allowing the utilization of spatial information. To enable spatial convolutions within $l_{ø}^{2}$, we transformed the data between network components using deterministic reshaping operations. More formally, let:

- $R_{\text{reshape},1}:{\mathbb{R}}^{B\times N\times h\times w\times 2}\to {\mathbb{R}}^{P\times N\times 2}$ be a reshaping operation that reorganizes a batch of B patches with spatial structure h×w and sequence length N (number of *b* values) into a batch of P=B×h×w voxels with sequence length N.
- $l_{ø}^{1}:{\mathbb{R}}^{P\times N\times 2}\to {\mathbb{R}}^{P\times M}$ be the first neural network, which maps each sequence of N×2 to a hidden state of size M (M=128).
- $f_{ø}$: ${\mathbb{R}}^{P\times M}\to {\mathbb{R}}^{P\times M}$ be the second neural network, which evolves the hidden state.
- $R_{\text{reshape},2}:{\mathbb{R}}^{P\times M}\to {\mathbb{R}}^{B\times M\times h\times w}$ be a reshaping operation that reorganizes the batch of P hidden states of size M into B patches (with 2D spatial dimensions h and w, and the dimension of the hidden state M) suitable for convolutional processing.
- $l_{ø}^{2}:$ ${\mathbb{R}}^{B\times M\times h\times w}\to {\mathbb{R}}^{B\times 4\times h\times w}$ be the third neural network (in our case a UNet), which reads out a batch of spatially structured hidden states (M×h×w) into four maps of four IVIM MRI parameters (h×w).

**FIGURE 1:**
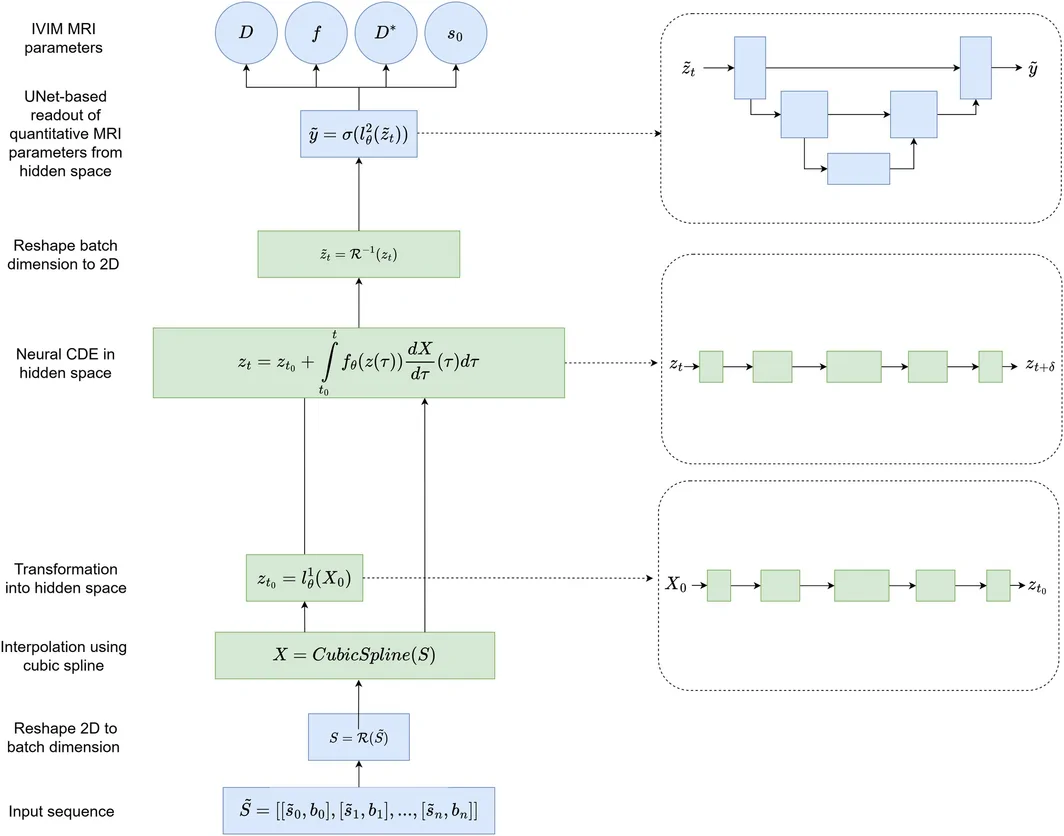
Schematic representation of a Spatial NCDE. The Spatial NCDE is composed of three neural networks: $l_{ø}^{1}$, $f_{ø}$, and $l_{ø}^{2}$. The input sequence $\tilde{S}$ is a 3D (2 spatial axes plus a *b*‐value axis) array, the *b*‐value axis can have an arbitrary length and irregular sampling intervals, the spatial axes can take any integer value n such that $\frac{n}{4}$ is also an integer. Spatially structured arrays are denoted with a tilde, 1D arrays are not ($\tilde{S}$ vs. S). Operations that require spatially structured arrays as input are shown in blue, operations that require voxel‐wise arrays as input are shown in green. Both $l_{ø}^{1}$ and $f_{ø}$ evaluate input on a voxel‐wise basis, the output of $f_{ø}$ is reshaped from the batch axis to spatial dimensions. Figure is adapted from our previous, voxel‐wise work [22].

$R_{\text{reshape},2}$ is the inverse of $R_{\text{reshape},1}$. For a detailed specification of the Spatial NCDE architecture, refer to Table S1.

Spatial NCDEs were trained to map the input sequence to four output parameters ($D,D^{*},f,s_{0}$). To constrain the estimates, network outputs were treated as coefficients (${\hat{y}}_{\text{parameter},\text{coeff}}$) which were rescaled to pre‐specified parameter‐specific ranges $\left(y_{\text{parameter},\min},y_{\text{parameter},\max}\right)$ ([0.0003−0.0035] $\frac{{\mathrm{mm}}^{2}}{s}$ for D, [0.005−0.1] $\frac{{\mathrm{mm}}^{2}}{s}$ for $D^{*}$ and [0−0.75] for f) using sigmoid operators [22].

Spatial NCDEs were trained by minimizing a supervised loss (L) in simulated data, following:

(2) $$L=\frac{1}{4}\sum_{m=1}^{4}{\left(\sigma \left({\hat{y}}_{m,\text{coeff}}\right)-\sigma \left(y_{m,\text{coeff}}\right)\right)}^{2},$$

where $\sigma \left({\hat{y}}_{m,\text{coeff}}\right)$ and $\sigma \left(y_{m,\text{coeff}}\right)$ represent the *m*‐th elements of the range‐normalized IVIM parameter estimates and range‐normalized ground truth IVIM parameters, respectively.

In vivo, no ground truth parameter information is available, and therefore a self‐supervised loss ($L_{\text{self}-\text{supervised}}$) was minimized to train a model pretrained on simulated data, following:

(3) $$L_{\text{self}-\text{supervised}}=\frac{1}{\mathrm{len}\left(s_{n,\text{input}}\right)}\sum_{n=1}^{\mathrm{len}\left(s_{n,\text{input}}\right)}{\left(s_{n,\text{input}}-s_{n,\text{pred}}\right)}^{2},$$

where $s_{n,\text{input}}$ represents the *n*‐th element of the normalized input signal and $s_{n,\text{pred}}$ represents the *n*‐th element of the modeled signal curves, calculated based on the predicted parameters and Equation (1). Note that using NCDEs (both 1D NCDE and Spatial NCDE) allows n to be variable for different elements inside a batch.

#### 2.2.2 Reference Method: 1D NCDE

As a reference without spatial awareness, we trained the 1D NCDE [22] that used a similar framework as the proposed Spatial NCDE. The key difference between the 1D NCDE and the Spatial NCDE was the architecture of $l_{ø}^{2}$: the 1D NCDE used parallel MLPs to read out the hidden state. The input sequence contained pairs of DWI signal intensities and the *b*‐values at which these signals were measured ($S_{i}=\left[s_{i},b_{i}\right]$). All computations remained in 1D feature space throughout. Parameter bounds, rescaling, signal modeling, and loss functions were identical to the Spatial NCDE implementation.

#### 2.2.3 Reference Method: UNet

As a reference without acquisition independence, we trained a UNet [20]. UNets exploit spatial correlations in measured signal through convolutional processing, bypassing the hidden state evolution characteristic of NCDEs. The input sequence ($\tilde{s}$) only contained the spatially structured DWI signal intensities. Parameter bounds, rescaling, signal modeling and loss functions were identical to the Spatial NCDE implementation.

Note that, as UNets are acquisition‐specific, we trained multiple UNets over the following experiments, one for each acquisition setting.

#### 2.2.4 Reference Methods: LSQ Fitting and s‐egmented Fitting

As a reference for conventional model fitting, two algorithms were used: LSQ fitting to all parameters at once and a segmented fitting approach (as recently suggested in the IVIM consensus manuscript [25]). The LSQ fitting was based on the optimize.curve_fit() function of the Scipy (https://docs.scipy.org/doc/scipy/, v1.13.1) package, using the trust‐region reflective algorithm with the following bounds ([lower, upper]): [0.0003,0.0035] $\frac{{\mathrm{mm}}^{2}}{s}$, [0.005,0.1] $\frac{{\mathrm{mm}}^{2}}{s}$ and [0,0.75], for D, $D^{*}$, and f, respectively. The segmented fitting used the TF_reference_IVIMFfit implementation by OSIPI (https://github.com/OSIPI/TF2.4_IVIM-MRI_CodeCollection) with the same bounds.

### 2.3 Data

#### 2.3.1 Simulations

To evaluate model performance in a controlled setting, with known ground truths, we performed simulations. For networks to exploit spatial awareness, parameter maps need intra‐parameter coherence (neighboring voxels with similar values) and inter‐parameter coherence (characteristic combinations of physiological parameters per tissue), without creating consistent relations between parameter values which would oversimplify the learning task and misrepresent in vivo conditions. Tissue boundaries are also often shared between physiological parameters. To achieve this, we used Perlin noise [26, 27] to create a base map partitioned into regions, each simulating a distinct tissue type with unique parameter ranges. Within each region, voxel values combined shared and parameter‐specific structure to ensure spatial dependence and controlled inter‐parameter correlation, producing realistic, heterogeneous parameter distributions (full procedure in Supporting Information S2.1.3, and an example in Figure 2, left column). These spatially correlated parameter patches were used as input to generate signal curves using Equation (1), with *b*‐values sampled as described in Table 1.

**FIGURE 2:**
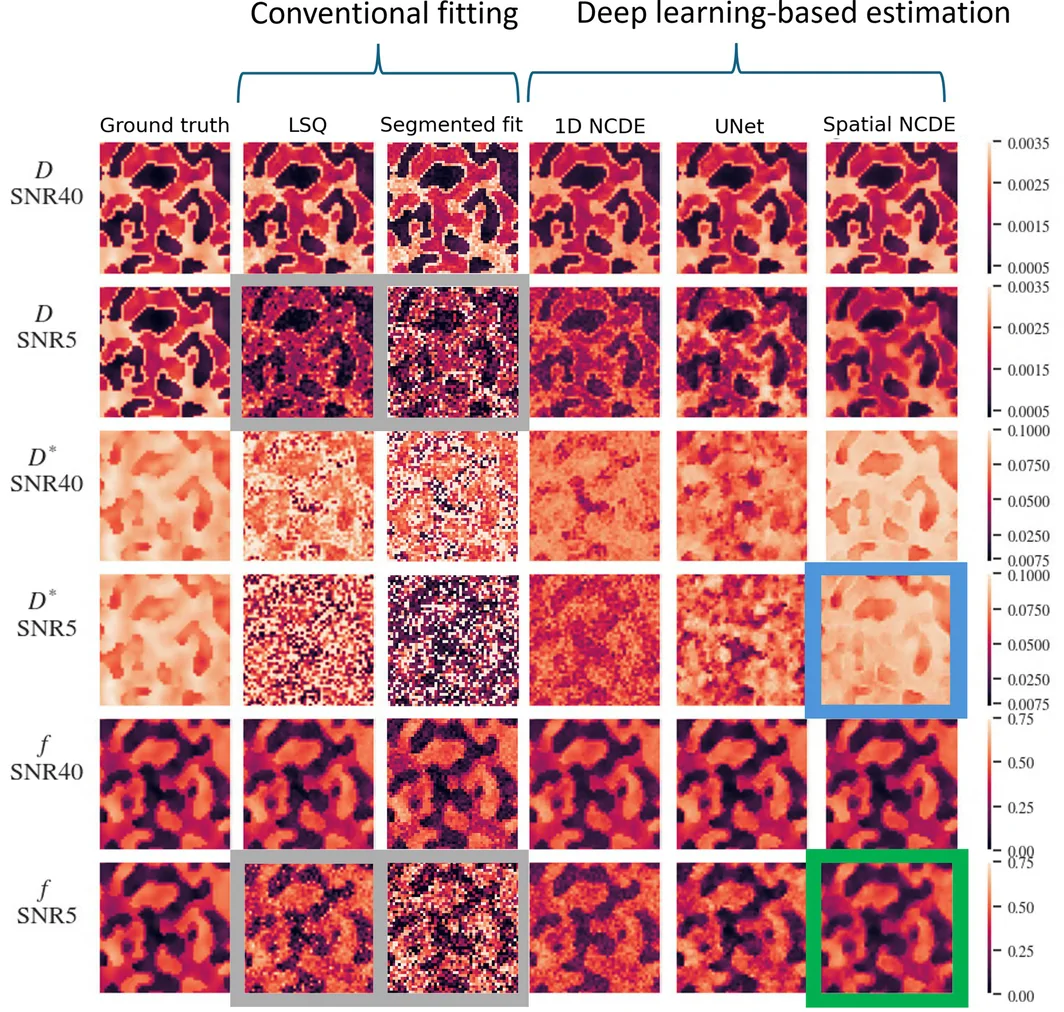
Simulated ground truth and estimated parameter maps for IVIM parameters at high (SNR40) and low (SNR5) SNR levels. Columns from left to right: Ground truth parameter maps, LSQ parameter maps, segmented fit parameter maps, UNet parameter maps, 1D NCDE parameter maps, Spatial NCDE parameter maps. Rows from top to bottom: Diffusion coefficient maps at SNR 40, diffusion coefficient maps at SNR 5, pseudo‐diffusion coefficient maps at SNR 40, pseudo‐diffusion coefficient maps at SNR 5, pseudo‐diffusion signal fraction maps at SNR 40, pseudo‐diffusion signal fraction maps at SNR 5. For parameter estimation at SNR 5, the Spatial NCDE‐based maps showed the highest resemblance to the ground truth maps across all parameters and across all estimation methods. Compared to ground truth parameter maps (left column), LSQ‐based and segmented fitting‐based maps (gray squares) had a noisier appearance than the other estimation methods. For estimation of the pseudo‐diffusion coefficient at SNRs 5 and 40 (blue square) and the pseudo‐diffusion signal fraction at SNR 5 (green square), the Spatial NCDE‐based map showed the highest resemblance to the ground truth maps compared with all estimation methods. For estimation of the diffusion coefficient and pseudo‐diffusion signal fraction at SNR 40, all estimation methods produced highly similar maps.

**TABLE 1:** Description of the protocol for sampling the *b*‐values.

Data type	Data usage	Acquisition protocol	Sequence length	*B*‐values sampled
Simulation	Training of: − 1D NCDE − Spatial NCDE	Variable	[5,17]	Given B=[0,1,2,5,10,20,30,40,50,75,100,150,200,300,400,500,600,700] $\frac{s}{{\mathrm{mm}}^{2}}$ N is uniformly distributed on [5,6,7,…,17] For N *b* values: Sample *b*‐value of $0\;\frac{s}{{\mathrm{mm}}^{2}}$ Randomly sample $\left\lceil \frac{N\;}{2}\right\rceil -1$ *b*‐values <100 $\frac{s}{{\mathrm{mm}}^{2}}$ from B Randomly sample $\lfloor \frac{N}{2}$⌋ *b‐*values ≥100 and $<700\;\frac{s}{{\mathrm{mm}}^{2}}$ from B
	Training of: − UNet Evaluation of: − LSQ − Segmented fitting − 1D NCDE − UNet − Spatial NCDE	Fixed	5	$\left[0,5,20,50,400\right]\;\frac{s}{{\mathrm{mm}}^{2}}$
		Fixed	9	$\left[0,1,5,10,20,50,100,400,700\right]\;\frac{s}{{\mathrm{mm}}^{2}}$
		Fixed	14	$\left[0,1,2,5,10,20,30,50,100,150,300,400,500,700\right]\frac{s}{{\mathrm{mm}}^{2}}$
		Fixed	18	[0,1,2,5,10,20,30,40,50,75,100,150,200,300,400,500,600,700] $\frac{s}{{\mathrm{mm}}^{2}}$
In vivo	Training of: − 1D NCDE − Spatial NCDE	Variable	[7,13]	Given B=[0,10,20,30,40,50,75,100,200,350,500,650, 800] $\frac{s}{{\mathrm{mm}}^{2}}$ N is uniformly distributed on [7,8,9,…,13] For N *b‐*values: Sample *b*‐value of $0\;\frac{s}{{\mathrm{mm}}^{2}}$ Randomly sample $\left\lceil \frac{N\;}{2}\right\rceil -1$ *b‐*values <100 $\frac{s}{{\mathrm{mm}}^{2}}$ from B Randomly sample $\lfloor \frac{N}{2}$⌋ *b*‐values ≥100 and $<700\;\frac{s}{{\mathrm{mm}}^{2}}$ from B
	Training of: − UNet Evaluation of: − LSQ − Segmented fitting − 1D NCDE − UNet − Spatial NCDE	Fixed	13	[0,10,20,30,40,50,75,100,200,350,500,650, 800] $\frac{s}{{\mathrm{mm}}^{2}}$
	Evaluation of: − LSQ − Segmented fitting − 1D NCDE − Spatial NCDE	Fixed	9	[0,10,20,30,50,100,200,350, 800] $\frac{s}{{\mathrm{mm}}^{2}}$

*Note:* These sampling procedures have been used during training and evaluation. The ceiling operator (⌈x⌉) rounds x up to the nearest integer, the floor operator (⌊x⌋) rounds x down to the nearest integer.

Rician noise was added to the simulated signal by adding Gaussian noise to the real and complex plane and taking the magnitude image. The signal‐to‐noise ratio of the signals were set to a predefined value (${\mathrm{SNR}}_{\text{predefined}}$, with range [5,50] in training data and [5,40] in evaluation data) and reflected SNR at =0 $\frac{s}{{\mathrm{mm}}^{2}}$.

#### 2.3.2 In Vivo

IVIM MRI data of esophageal cancer were acquired in 45 patients using a 1.5 T Philips MRI scanner (Ingenia/Achieva, Philips, Best, the Netherlands) at the UMC Utrecht, a single‐center subset of the PRIDE multicenter study [28] All patients over 18 with newly diagnosed, biopsy‐proven esophageal adeno‐ or squamous cell carcinoma scheduled for neoadjuvant chemoradiotherapy (nCRT) and surgery between August 2017 and December 2022 were eligible. Exclusion criteria included MRI contraindications and prior endoscopic mucosal resection or endoscopic submucosal dissection for the primary tumor before nCRT. Neoadjuvant chemoradiotherapy consisted of weekly administration of carboplatin and paclitaxel combined with 23 fractions of radiotherapy over 5 weeks. After surgery, tumor response was assessed by gastrointestinal pathologists where patients with 0% residual tumor were categorized as pathological complete response (pCR) and other patients were categorized as pathological non‐complete response (non‐pCR). During the study, MR imaging was acquired three times: before nCRT (MRI‐1), during week 3 of nCRT (MRI‐2) and before surgery (MRI‐3). Sagittal DW‐MRI was acquired in free breathing using 13 *b*‐values between 0 and 800 S/mm^2^ (Table 1). Detailed MRI acquisition settings are provided in Table S1. The study was approved by the local medical ethics review committee. All participants provided written informed consent, and research was performed in accordance with the Declaration of Helsinki guidelines. Only patients with complete MRI datasets were included in the present analysis (except for one patient where MRI‐2 was not acquired). Preprocessing is described in Section S2.1.4 [29].

### 2.4 Experiments

#### 2.4.1 Training and Evaluation on Simulation Data

##### 2.4.1.1 Training on Simulation Data

All simulated training and evaluation data were generated on the fly, per Section 2.3.1, using *b*‐values sampled from the fixed (UNet) or variable (1D NCDE, Spatial NCDE) configurations in Table 1. The Adam optimizer was used for training [30].

The Spatial NCDE trained for 750 epochs (100 training and 50 validation batches per epoch, batches of 32 patches of 16 by 16 voxels). Learning rate started at $1\times 10^{-4}$, and halved at epochs 80, 150, 250, 350, and 500. An adaptive step solver kept the solution error within a predefined tolerance [23], with absolute and relative tolerance set to 0.00001 and 0.001, respectively, and halved at epochs 50, 80, 150, and 250. After 750 epochs, the model was selected from the epoch with the lowest validation loss.

The 1D NCDE was trained similarly to the Spatial NCDE; however, training and validation batches only contained 1D data.

The UNet was trained similarly to the Spatial NCDE, but with fixed *b*‐value sets.

##### 2.4.1.2 Evaluation of Simulated Data

IVIM MRI parameter estimates were evaluated on simulated signal curves generated in accordance with Section 2.3.1, using *b*‐values sampled according to the fixed acquisition protocols described in Table 1. For each of 5 SNR levels (SNR 5,10,20,30,and40) 1000 2D maps (size: [48,48,1]) were simulated (yielding a total of 11 520 000 simulated voxels). Since for these simulations the ground truth IVIM MRI parameter values were known, the mean squared error (MSE) per map was calculated using:

(4) $$\text{Mean squared error}=\frac{1}{N}\sum_{i=1}^{n}{\left({\hat{y}}_{i}-y_{i}\right)}^{2}$$

where $\hat{y}$ denotes the estimated IVIM parameter and y the ground truth IVIM parameter (with N=48×48). In addition, the mean, median, 25th and 75th percentiles of the MSEs over the 5000 parameter maps were calculated. The overall MSE functioned as a measure of accuracy, effect sizes were computed as a relative improvement in MSE for Spatial NCDE compared to the reference methods. The MSEs were decomposed into their respective bias and variance components. Pairwise comparisons between Spatial NCDE‐based MSEs and the reference methods were performed using Wilcoxon signed‐rank tests with Holm‐Bonferroni correction (*n* = 60) [31].

#### 2.4.2 Training and Evaluation In Vivo

##### 2.4.2.1 Training on In Vivo Data

The 1D NCDE, UNet and Spatial NCDE were trained to estimate IVIM parameters using a four‐fold cross validation. Cross‐validation meant all data was split at patient‐level into four different groups. Per fold, a model was trained on three groups and evaluated on the held‐out group. No test‐patient data contributed to model selection or normalization. The model trained on simulation data was used as initialization for in vivo training. Training lasted for 250 epochs (100 batches of 32 elements). The *b*‐values and corresponding signals for training and validation data were sampled as described in Table 1, using *b*‐values from the fixed (UNet) or variable (1D NCDE, Spatial NCDE) configurations. In vivo, the validation loss was too noisy to base the optimal model on, instead training duration was fixed at 250 epochs. The learning rate was fixed at $1\times 10^{-5}$. For the 1D NCDE and Spatial NCDE, absolute and relative error tolerances were set to 0.00001 and 0.001, respectively and halved at epochs 50, 80, and 150.

##### 2.4.2.2 Evaluation of In Vivo Data

For each deep learning‐based estimation method, evaluations were only performed on patients of the held‐out group for each of the four folds. In previous work using this data [32, 33], pathologic complete responders (pCR) were best distinguished from non‐complete responders (non‐pCR) using the changes in mean apparent diffusion coefficient (ΔADC) between MRI‐1 (before nCRT) and MRI‐2 (2 weeks into nCRT). Similar to that approach, in this work we quantified the apparent area under the ROC curve (AUROC), for distinguishing pCR from non‐pCR, using changes in median parameters of the esophageal tumor (ΔD, Δf, $\Delta D^{*}$) from MRI‐1 to MRI‐2. The 95% confidence interval of the AUROC was calculated using 1000 bootstrap samples. Statistical significance of the difference between the Spatial NCDE‐based AUROC and the AUROC of the reference methods was assessed using DeLong's test [34]. The tumor region (without the lumen) was manually delineated based on the signal of the baseline DW‐MRI with *b*value 500 s/mm^2^ [32] and a 2‐mm isotropic shrinkage margin was applied to exclude tumor boundary edges. The delineation was propagated to subsequent DW‐MRI scans using deformable b0‐b0 registration (VoxAlign, MIM Software, Cleveland, Ohio, USA) as described in [28].

Qualitative estimation performance was assessed by comparing parameter maps of the full field‐of‐view and zoomed‐in tumor regions visually and comparing the squared residuals of the reconstructed signal of the zoomed‐in tumor regions. To demonstrate the independence of the proposed method to variations in the acquisition protocol, parameter estimations were repeated with 9 of the available 13 *b‐*values and qualitatively compared to the estimations based on the full acquisition protocol.

## 3 Results

### 3.1 Simulations

Qualitatively, Spatial NCDEs best resembled ground truth maps for all IVIM parameters (Figure 2). Improvements compared to the other estimation methods became most apparent at SNR 5 for D and f, where Spatial NCDEs estimated less noisy maps, and for the hardest‐to‐estimate parameter, $D^{*}$, where Spatial NCDE was the only method estimating a correct map structure.

These qualitative findings were also reflected in the quantitative results. For 14 out of 15 comparisons across 3 IVIM parameters and 5 SNR levels, Spatial NCDEs had the lowest mean, median, 25th and 75th percentile of the MSE per simulated parameter map (Figure 3 and Table S3, exception $D^{*}$ at SNR 5, for which 1D NCDE had a better estimate). The improvements compared to other estimation methods (LSQ, segmented fitting 1D, NCDE, and UNet) were larger for harder‐to‐estimate parameters (f and $D^{*}$) and at lower SNR. Spatially aware networks (UNet and Spatial NCDE) estimations tended to result in lower MSEs than their voxel‐wise counterparts (1D NCDE). Similarly, Spatial NCDE consistently had lower MSEs than the acquisition‐specific counterpart, UNet. When sequence length and configuration of *b‐*values varied, the Spatial NCDE also had the lowest MSEs of all estimation methods for all 3 parameters, all 5 SNR levels and all 4 *b*‐value sets (Figure S3). The variance of the parameter estimation error per simulated map (Figure S4) showed a similar trend, where the Spatial NCDE‐based estimates had the lowest variance while segmented fitting‐based estimates had the highest variance. In contrast, the magnitude of the bias (Figure S4) was highest for both Spatial NCDE‐based estimates and LSQ‐based estimates for $D^{*}$ (SNR < 30) and f (all SNR levels).

**FIGURE 3:**
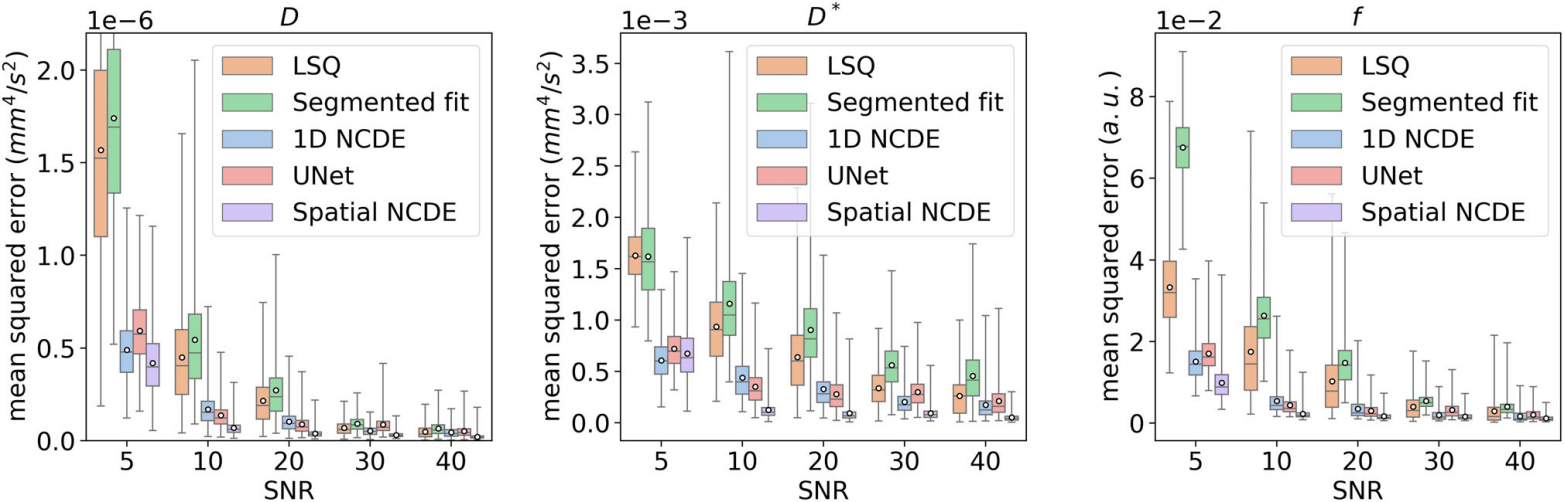
Box plots of the distribution of MSE errors over 1000 simulated parameter maps for D (left), $D^{*}$ (middle), and f (right) as a function of SNR for LSQ (orange), segmented fit (green), 1D NCDE (blue), UNet (pink) and Spatial NCDE (purple). The white circle represents the overall MSE over the simulated parameter maps. Across parameters and SNR levels, the Spatial NCDE consistently showed lower MSE compared to all other estimation methods. *B*‐values used for evaluation were [0, 1, 2, 5, 10, 20, 30, 40, 50, 75, 100, 150, 200, 300, 400, 500, 600, 700] $\frac{s}{{\mathrm{mm}}^{2}}.$ Figure S3 shows similar results for different *b*‐value sets. All differences were statistically significant, *p* values and effect sizes are reported in Table S3.

At inference, Spatial NCDEs ran substantially faster than conventional LSQ or segmented fitting, reaching a clinically reasonable time of 24 000 voxels/s (3.7 times faster than LSQ and 5 times faster than segmented fitting), 224 × 224 voxels per 2.1 s (Table S4). During training, the time required for one iteration (forward pass + backpropagation) did increase from UNet (2.22 s) to 1D NCDE (9.07 s) and Spatial NCDE (41.03 s) (Table S4). Both solving the controlled differential equation and a 2D convolution‐based mapping caused an increase in time required per iteration.

### 3.2 In Vivo Data

In patients with esophageal cancer, parameter maps generated by Spatial NCDEs showed similar structure and parameter values as parameter maps estimated using LSQ, but with less spatial variations that contain a noisy appearance (Figures 4a,b, S5 and S6). In particular, LSQ‐based maps tended to demonstrate a stronger tendency to estimate parameter values toward the edge of the allowed range (Figure 4b, $D^{*}$). This behavior is consistent with reduced parameter identifiability, increased sensitivity to noise, or convergence to local minima when using LSQ. Moreover, LSQ tended to find two solution modes in neighboring voxels, with mode 1: medium/high D, low f, medium/high $D^{*}$, and mode 2: low D, high f, low $D^{*}$ (Figure 4b, D and f, white dotted ovals). While the differences are smaller in the easiest‐to‐estimate parameter (D), the differences become more apparent for harder‐to‐estimate parameter f and particularly $D^{*}$ (Figure 4c, f and $D^{*},\text{black dotted ovals}$), uncovering a structure in the parameter map at Spatial NCDEs which remained hidden using LSQ ($D^{*}$). Even when the difference in parameter estimates was large (Figure 4c, f and $D^{*}$, black dotted ovals), differences in the signal reconstruction error were small (Figures 4d and S7). The signal reconstruction error was lower in some voxels for the LSQ‐based parameter estimates (Figures 4d and S7, red dotted ovals) and in other voxels for the Spatial NCDE‐based parameter estimates (Figures 4d and S7, blue dotted ovals). Spatial NCDEs appeared to estimate parameter maps that minimized both the spatial variation inside the maps and the signal reconstruction error inside each voxel. Comparing the Spatial NCDE‐based parameter maps to reference parameter maps shows overall similar structural features and parameter values (Figures S5–S7). Segmented fit yielded parameter maps for D that were smoother compared to those estimated by LSQ, 1D NCDE, UNet and Spatial NCDE. Without retraining, the Spatial NCDE produced similar maps for 9 *b*‐values as for 13 *b*‐values (Figure S5).

**FIGURE 4:**
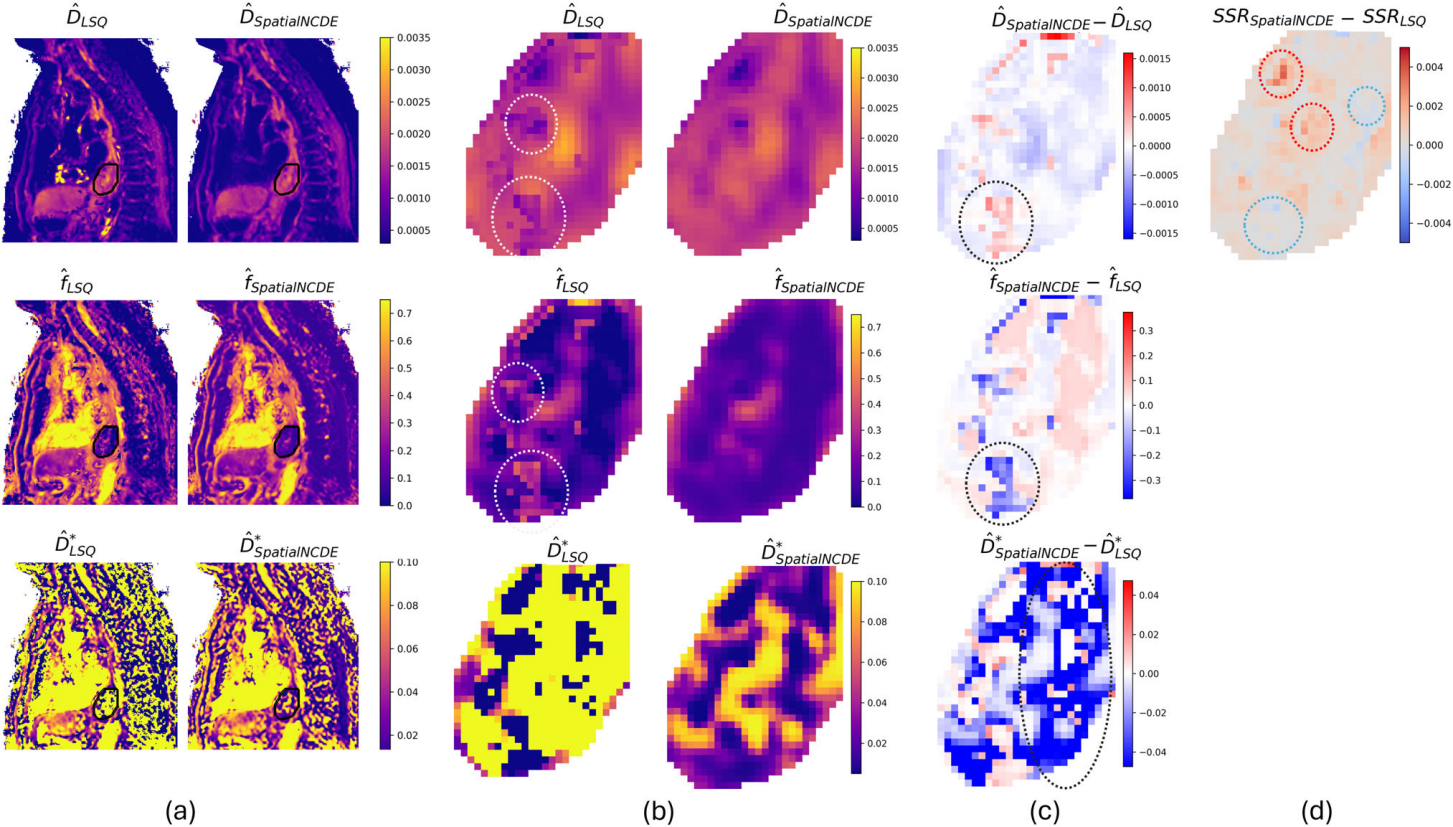
(a) parameter maps of the entire field‐of‐view in patients with esophageal cancer, with the tumor region outlined in black. Parameters were estimated using LSQ (${\hat{D}}_{\mathrm{LSQ}},{\hat{f}}_{\mathrm{LSQ}},\text{and}\;{\hat{D}}_{\mathrm{LSQ}}^{*}$, left column) and Spatial NCDE (${\hat{D}}_{\text{SpatialNCDE}},{\hat{f}}_{\text{SpatialNCDE}}\;\text{and}\;{\hat{D}}_{\text{SpatialNCDE}}^{*}$, right column). (b) Close‐up and masked views of the esophageal tumor, showing parameter maps estimated using both LSQ and Spatial NCDE. (c) Differences between the estimated parameter maps (Spatial NCDE minus LSQ). (d) Differences in signal reconstruction error, quantified as the sum of squared residuals (SSR), using the forward‐modeled signal from the LSQ‐based parameter estimates (${\mathrm{SSR}}_{\mathrm{LSQ}}$) and Spatial NCDE‐based parameter estimates (${\mathrm{SSR}}_{\text{SpatialNCDE}}$). In this figure, red voxels mean LSQ was more data consistent, whereas blue voxels mean Spatial NCDE was more data consistent. Overall, Spatial NCDE and LSQ produced parameter maps with similar structural features, while Spatial NCDE yielded spatially smoother maps (b, white dotted ovals). Although the differences in parameter estimates were substantial at some locations (c, black dotted ovals), differences in signal reconstruction error remained small (d). Regions where ${\mathrm{SSR}}_{\mathrm{LSQ}}$ was lower than ${\mathrm{SSR}}_{\text{SpatialNCDE}}$ are indicated by red dotted ovals, whereas regions where ${\mathrm{SSR}}_{\text{SpatialNCDE}}$ was lower than ${\mathrm{SSR}}_{\mathrm{LSQ}}$ are indicated by blue dotted ovals (d). Parameter maps of the entire field‐of‐view, close‐up and masked views of the esophageal tumor for segmented fitting, 1D NCDE and UNet are shown in Figures S5 and S6.

Out of 45 patients, 14 were pCR. The apparent AUROC of changes in IVIM parameters between baseline and 2 weeks into chemoradiotherapy for distinguishing pCR from non‐pCR did not differ significantly between Spatial NCDE and any of the reference methods for any IVIM parameter (Table 2). Although Spatial NCDE achieved numerically higher AUROC values than LSQ for 3/3 IVIM parameters (0.643 vs. 0.613 for D, 0.673 vs. 0.602 for f, and 0.569 vs. 0.567 for $D^{*}$), the 95% confidence intervals showed substantial overlap and the differences were not statistically significant (Table 2). Compared with the reference methods, Spatial NCDE also yielded higher apparent AUROC values than segmented fitting for 2/3 IVIM parameters, than 1D NCDE for 1/3 parameters and than UNet for 1/3 IVIM parameters, however none of these differences reached statistical significance.

**TABLE 2:** Apparent areas under the ROC curve (AUROC) and their 95% confidence interval for the discrimination between pCR and non‐pCR patients with esophageal cancer, computed using the difference in median value of the parameter maps at MRI‐1 and MRI‐2 estimated using all 13 *b*‐values with LSQ, segmented fit, 1D NCDE, UNet and Spatial NCDE.

Parameter		LSQ	Segmented fit	1D NCDE	UNet	Spatial NCDE
ΔD	Apparent AUROC (95% CI)	0.613 (0.417–0.790)	0.599 (0.397–0.797)	0.621 (0.429–0.792)	0.657 (0.458–0.828)	0.643 (0.438–0.812)
	*p* value	0.59	0.53	0.69	0.77	—
Δf	Apparent AUROC (95% CI)	0.602 (0.419–0.774)	0.739 (0.569–0.882)	0.673 (0.489–0.828)	0.621 (0.430–0.801)	0.673 (0.488–0.845)
	*p* value	0.20	0.29	1	0.10	—
$\Delta D^{*}$	Apparent AUROC (95% CI)	0.566 (0.371–0.747)	0.558 (0.349–0.753)	0.665 (0.472–0.838)	0.558 (0.362–0.747)	0.569 (0.368–0.761)
	*p* value	0.96	0.91	0.11	0.71	—

*Note:* The *p* value is derived from the DeLong's test between the apparent AUROC of the Spatial NCDE and the reference method, respectively.

## 4 Discussion

In this study, we demonstrate that incorporating spatial awareness into NCDEs improves the estimation of IVIM MRI parameters while preserving their generalization capabilities. We are the first to combine spatial awareness and acquisition‐independence in QMRI parameter estimation in general, and for IVIM parameter estimation in specific. Our Spatial NCDE outperformed LSQ, segmented fitting, UNet and our 1D NCDE for a broad range of SNRs and acquisition protocols in simulated data both quantitatively and qualitatively. In addition, Spatial NCDEs demonstrated generalizability to in vivo data by estimating IVIM parameter maps that were consistent with parameter maps obtained using LSQ, segmented fitting, 1D NCDEs and UNet. Compared to LSQ ($D^{*}$, D, and f) and segmented fitting ($D^{*}$), the Spatial NCDE‐based maps were less sensitive to noise and appeared less prone to polarized parameter estimates. The in vivo maps produced by the Spatial NCDE were very similar to those from the UNet. However, unlike the UNet, the Spatial NCDE was able to generalize to arbitrary *b*‐value combinations, highlighting its flexibility. This evaluation does not provide evidence of improved clinical predictive performance relative to reference methods. However, the qualitative in vivo results combined with the quantitative and qualitative simulation results suggest that Spatial NCDEs are able to combine the beneficial properties of neural networks with the generalizability and ease‐of‐use of conventional methods, making it feasible to implement a single pre‐trained Spatial NCDE for most applications. This removes a large hurdle in bringing spatially aware neural networks to a broad audience.

In simulations, Spatial NCDEs achieved lower MSEs than all other parameter estimation methods across acquisition protocols and SNR levels. While 1D NCDEs already outperformed LSQ fitting for low SNR levels and short acquisition protocols [22], Spatial NCDEs also outperformed LSQ for longer acquisitions and high SNR levels. In addition, Spatial NCDEs achieved lower MSE compared to 1D NCDEs across acquisition protocols and SNR levels. These results confirm findings in the literature that spatial awareness allows models to estimate IVIM parameters with higher accuracy by using information from surrounding voxels during estimation [20, 21]. Qualitative analysis of parameter maps confirmed the quantitative results, where the Spatial NCDE was the only estimation method able to estimate parameter maps visually similar to the ground truth under conditions of low SNR or for hard‐to‐estimate parameters ($D^{*},f$). Interestingly, Spatial NCDEs outperformed their acquisition‐specific counterpart, UNets, in simulations across acquisition protocols, even though UNets only needed to learn one specific mapping from a single set of *b*‐values to IVIM parameters. This suggests that modeling signal dynamics across *b‐*values may provide improved robustness to acquisition variability compared with learning protocol‐specific signal‐to‐parameter mappings. A possible explanation could be that controlled differential equations implicitly regularize the learning process by enforcing smoothness and temporal consistency in the model's hidden state evolution, which discourages overfitting to noise‐ and artifact‐induced input patterns. Improvements in the accuracy of (Spatial) NCDEs often came at the cost of an increase in the bias of the error, which confirms previous findings in the literature [22, 35]. However, for Spatial NCDE the magnitude of the bias was orders of magnitudes smaller than typical parameter values. Nonetheless, implementations to control the trade‐off between bias and variance of the error and to move deep learning‐based estimators closer to statistically efficient estimators exist and should be included in future implementations [35].

Although there is no ground truth available in vivo, there are indications that suggest that Spatial NCDEs yield more consistent, reliable parameters than LSQ. First, Spatial NCDE estimated parameter maps which appear to be more consistent with other reference methods than the LSQ counterparts. Second, LSQ‐based maps and segmented fitting‐based maps show large regions where $D^{*}$ hits the fit boundary, a result that suggests reduced parameter identifiability or increased sensitivity to noise. Third, LSQ tended to find multiple solution modes in neighboring voxels, a sign of parameter degeneracy. An example of this behavior may arise in data where the bi‐exponential behavior is less clearly pronounced and LSQ is either capturing the decay with $D^{*}$ or with D. Even though the objective of LSQ fitting is to minimize the voxel‐wise signal reconstruction error, a substantial part of the voxels in the tumor region had lower signal reconstruction error for the Spatial NCDE‐based parameter maps. This indicates that the spatial variations in LSQ‐based parameter maps are not reflections of underlying physiology, but side‐effects of parameter optimization in a noisy context.

Both the quantitative and qualitative results suggest that Spatial NCDEs effectively translate their parameter estimation capacity from simulated to in vivo data, yet the relative performance advantage seen in simulations does not carry over to the in vivo setting to the same extent. A key difference to simulations is that in vivo no ground truth is available, which precludes direct comparison between Spatial NCDEs and reference methods. We therefore evaluated the estimated biomarkers against clinical outcomes instead. Spatial NCDEs yielded slightly higher AUROCs than LSQ fitting for predicting pCR after nCRT in esophageal cancer across all three IVIM parameters, although none of these differences were statistically significant. Compared to segmented fitting, 1D NCDEs, and UNets, however, no improvement in apparent AUROC was observed for Spatial NCDE‐based parameter estimates. This contrasts with the substantial differences in relative performance between Spatial NCDE and the other estimation methods observed on simulated data. This observed decrease in relative performance when moving from simulated data to in vivo data could stem from several factors. First, the loss function differed between training on simulations and in vivo (supervised in simulations, self‐supervised in vivo) and may lead to a different parameter estimation efficacy. Second, as part of the standard preprocessing pipeline in this study, the in vivo data was denoised to facilitate more reliable conventional fitting, moving to a higher SNR regime in which the differences between methods become smaller. This denoising is required to get sensible LSQ maps but potentially comes at the cost of introducing regularization bias. Fourth, in the absence of ground truth, we relied on indirect clinical outcome measures, which are a noisier proxy for estimation accuracy than a direct comparison would be. In general, AUROC for clinical outcome prediction depends not only on the accuracy of parameter estimation but primarily on the existence and strength of the relationship between the biomarker and the clinical outcome. For the specific case of esophageal cancer and diffusion‐based biomarkers, this relationship remains under debate [28, 29, 30, 31].

Although Spatial NCDEs have substantial potential, several requirements may limit applicability. They share the limitations of 1D NCDEs: input signal normalization is required, long input sequences make training slower and less stable, and the parameter‐rescaling range must match between training and inference [22, 36, 37]. Because Spatial NCDEs exploit spatial dependencies learned from the training data, performance may degrade when spatial patterns at inference differ substantially. NCDEs also require more computation per backpropagation than UNets, especially in the spatial setting, making them slower to train. During inference, however, Spatial NCDEs process whole patches at once, yielding estimation speeds that are substantially higher than LSQ and segmented fitting, and comparable to 1D NCDEs.

Several aspects were not studied in detail in this proof‐of‐principle research. For example, the robustness of Spatial NCDEs to common DWI‐related artifacts, such as motion, eddy currents, and susceptibility‐induced distortions, was not explicitly evaluated. Although incorporating spatial information may improve robustness to random noise, systematic spatial artifacts may also affect neighboring voxels and should therefore be minimized using standard diffusion MRI preprocessing, including motion, eddy‐current, and distortion correction, before parameter estimation. Moreover, the proposed approach only provides point estimates of the IVIM parameters and does not quantify voxel‐wise or region‐wise uncertainty [38, 39]. Finally, we did not evaluate the robustness of Spatial NCDEs to the assumed spatial structure learned during training. While our simulations go beyond simple Perlin noise, using distinct tissue types with sharp boundaries and gradually varying, non‐correlated parameter values, how sensitive Spatial NCDEs are to deviations between learned and true spatial structure remains an open question.

Successful application of QMRI to clinical use‐cases has been hindered by both poor accuracy in parameter estimation and difficulty in adopting specialized algorithms. For QMRI parameter estimation, accuracy and ease‐of use appear to be at odds with each other, where complex algorithms require substantial effort to set up and simple algorithms are not effective enough in the complex environments of modern‐day imaging studies. We believe that Spatial NCDEs are a step toward integrating both requirements, where versatile networks that exhibit robustness to varied acquisition protocols can enable end users to directly apply a pre‐trained, validated model which has been developed and validated in a dedicated environment to analyze highly specialized data in a fast, accurate and reliable fashion.

## 5 Conclusion

Spatial NCDEs are a robust and versatile tool for IVIM MRI parameter estimation, offering the generalizability and ease‐of‐use of conventional methods alongside the improved accuracy of neural networks. Their ability to utilize spatial information results in more accurate and consistent parameter maps across a wide range of settings. This advancement paves the way for wider clinical and research adoption of IVIM MRI, allowing for reliable and efficient analysis across different imaging protocols without the need for retraining specialized models.

## Funding

This publication is part of the project “Self‐supervised physics‐informed deep learning quantitative MRI of tumour microstructural imaging for personalized cancer treatments” with file number 20367 of the VIDI research programme, which is financed by the Dutch Research Council (NWO). This work was also funded by the KWF Dutch Cancer Society (KWF‐UVA 2021.13785, O.J.G‐C. and D.K.).

## Conflicts of Interest

The authors declare no conflicts of interest.

## Supporting information


**Table S1:** Details of the architecture of the Spatial NCDE. A description of $l_{ø}^{1}$, $f_{ø}$, and $l_{ø}^{2}$ can be found in Section 2.2.1.
**Table S2:** Details of the acquisition protocol.
**Figure S1:** (a) Shared base map defining the global structure for all IVIM MRI parameters. (b) the shared base map partitioned into three distinct, equally sized regions.
**Figure S2:** (a) IVIM MRI parameter maps (D, $D^{*},$ and f, respectively) showing shared spatial structure. (b) 2D distribution plots showing no consistent inter‐parameter correlation based on parameter values.
**Section S2.1.4:** In‐vivo preprocessing.
**Figure S3:** Box plots of the distribution of MSEs over 1000 simulated parameter maps for D (top row), $D^{*}$ (middle row), and f (bottom row) as a function of SNR (columns from left to right: SNR 5, 10, 20, 30, 40) and as a function of sequence length (x‐axes) for LSQ (orange), segmented fit (green), 1D NCDE (blue), UNet (pink), and Spatial NCDE (purple). *B* values used for evaluation can be found in Table 1. The segmented fit could not be performed on 5 *b* values, as the algorithm required more *b* values for the multi‐step approach. The white circle represents the MSE over simulated parameter maps. Across parameters and SNR levels, the Spatial NCDE consistently showed lower MSEs compared to all other estimation methods. Note that the NCDE and Spatial NCDE were trained once on a dataset with variable *b* value configurations, whereas UNet was trained separately for each *b* value configuration.
**Figure S4:** Top row: Bar plot of the bias of the error of D (left column), $D^{*}$ (middle column) and f (right column) as a function of SNR for LSQ (orange), segmented fit (green), 1D NCDE (blue), UNet (pink) and Spatial NCDE (purple). Bottom row: Bar plot of the mean of the variance per simulated parameter map of estimated IVIM parameters as a function of SNR for LSQ (orange), segmented fit (green), 1D NCDE (blue), UNet (pink) and Spatial NCDE (purple).
**Table S3:** Overview of effect sizes and *p* values of the MSE in simulated data for Spatial NCDE compared to LSQ, segmented fit, 1D NCDE and UNet. Spatial NCDE outperformed all other estimation methods by estimating lower MSEs for 14/15 comparisons across parameters and SNR levels (except for 1D NCDE estimating lower MSE for $D^{*}$ at SNR5). All differences were significant (*p* value < 0.05). The effect size was computed as $\frac{{\mathrm{MSE}}_{\text{reference}}-{\mathrm{MSE}}_{\text{SpatialNCDE}}}{{\mathrm{MSE}}_{\text{reference}}}\times 100\%$.
**Table S4:** Overview of inference speed and training time per iteration for LSQ, segmented fit, 1D NCDE, UNet and Spatial NCDE. While the Spatial NCDE training was substantially slower than UNet, the inference speed was comparable to that of the 1D NCDE and substantially faster than LSQ and segmented fit. Spatial NCDE predicted a 224 × 224 slice in 2.05 s on an NVIDIA A100 GPU, reaching clinically feasible speeds.
**Figure S5:** Top half parameter maps of the close‐up views of the tumor region in patients with esophageal cancer. Parameters were estimated using LSQ, segmented fit, 1D NCDE, UNet and Spatial NCDE using all 13 available *b* values. Overall, Spatial NCDE, UNet, 1D NCDE and segmented fit produced parameter maps with similar structural features and parameter values, while LSQ produced parameter maps which were dominated by the upper bound of the parameter range ($D^{*}$), or yielded spatially inconsistent attributions of signal decay to either D or f (white dotted ovals). While Spatial NCDE, UNet, 1D NCDE and segmented fit yielded more consistent parameter estimates some differences were apparent. 1D NCDE estimated higher f values where $D^{*}$ was close to 0, and thus their product harder to define (blue dotted ovals). Segmented fit yielded parameter maps for D that were more smoothed compared to the estimations by LSQ, 1D NCDE, UNet and Spatial NCDE. Bottom half: Parameter maps of the close‐up views of the tumor region in patients with esophageal cancer estimated using only 9 of the available 13 *b* values to determine the robustness to variations to the acquisition protocol. The UNet was not evaluated, since it required retraining to be evaluated on a different acquisition protocol. LSQ, Overall, segmented fit, 1D NCDE and Spatial NCDE produced parameter maps which were largely consistent with their respective estimations using 13 *b* values (see top half). The most notable differences were that the estimated values for D became substantially higher throughout the parameter map for the 1D NCDE, and that both the segmented fit and LSQ had a larger portion of estimates on the upper bound of the parameter range for $D^{*}$.
**Figure S6:** Parameter maps of the entire field‐of‐view in patients with esophageal cancer. Parameters were estimated using LSQ, segmented fit, 1D NCDE, UNet and Spatial NCDE.
**Figure S7:** Top row: Difference in sum of squared residuals (SSR) of the close‐up views of the tumor region in patients with esophageal cancer between Spatial NCDE and LSQ, segmented fit, 1D NCDE and UNet, respectively. Red indicates an area where the reference method (either LSQ, segmented fit, 1D NCDE or UNet) has lower SSR than Spatial NCDE, while blue indicates an area where Spatial NCDE has lower SSR than the reference. Bottom three rows: Difference in parameter estimates between Spatial NCDE and LSQ, segmented fit, 1D NCDE and UNet, respectively. Between Spatial NCDE and LSQ, LSQ has spatially inconsistent assignments of signal decay to either D or f, while the Spatial NCDE‐based estimations are more spatially coherent. Compared to the segmented fit, Spatial NCDE show a pattern where attributions to signal decay are assigned more to either D or f, leading to difference maps for D and f which are roughly inversted. Compared to 1D NCDE, Spatial NCDEs parameter maps share the same structure but with a parameter‐specific offset. Finally, the parameter maps for Spatial NCDE and UNet are very similar, especially for D and f.

## Data Availability

Our code and parameters are available at: https://github.com/DKuppens/SpatialNCDE-QMRI.
