# Supplementary material for: Spatially Aware Acquisition‐Independent Deep Learning for IVIM MRI Parameter Estimation in Patients With Esophageal Cancer
Source: Magn Reson Med. 2026 Sep 14;97(1):365–77. doi: 10.1002/mrm.70592 (PMC13746104; doi:10.1002/mrm.70592)
Supplement: Supplementary file 1 [file MRM-97-365-s001.docx]

## SUPPORTING INFORMATION

*Description of supporting information*

Table S1: Details of the architecture of the Spatial NCDE.

Table S2: Details of the acquisition protocol.

Table S3: Overview of effect sizes and p-values of the MSE in simulated data for Spatial NCDE compared to LSQ, segmented fit, 1D NCDE and UNet. Spatial NCDE outperformed all other estimation methods by estimating lower MSEs for 14/15 comparisons across parameters and SNR levels (except for 1D NCDE estimating lower MSE for $D^{*}$ at SNR5). All differences were significant (p-value < 0.05). The effect size is computed as $\frac{{MSE}_{reference}-{MSE}_{SpatialNCDE}}{{MSE}_{SpatialNCDE}}x100\%$.

Table S4: Overview of inference speed and training time per iteration for LSQ, segmented fit, 1D NCDE, UNet and Spatial NCDE. While the Spatial NCDE training was substantially slower than UNet, the inference speed was comparable to that of the 1D NCDE and substantially faster than LSQ and segmented fit. Spatial NCDE predicted a 224 x 224 slice in 2.05 seconds on an NVIDIA A100 GPU, reaching clinically feasible speeds.

Fig. S1: a) shared base map defining the global structure for all IVIM MRI parameters. b) the shared base map partitioned into three distinct, equally sized regions.

Fig. S2: a) IVIM MRI parameter maps ($D$, $D^{*}$and $f$, respectively) showing shared spatial structure. b) 2D distribution plots showing no consistent inter-parameter correlation based on parameter values.

Fig. S3: Box plots of the distribution of MSEs over 1000 simulated parameter maps for $D$ (top row), $D^{*}$ (middle row) and $f$ (bottom row) as a function of SNR (columns from left to right: SNR 5, 10, 20, 30, 40) and as a function of sequence length (x-axes) for LSQ (orange), segmented fit (green), 1D NCDE (blue), UNet (pink) and Spatial NCDE (purple). B-values used for evaluation can be found in Table 1. The segmented fit could not be performed on 5 b-values, as the algorithm required more b-values for the multi-step approach. The white circle represents the MSE over simulated parameter maps. Across parameters and SNR levels, the Spatial NCDE consistently showed lower MSEs compared to all other estimation methods. Note that the NCDE and Spatial NCDE were trained once on a dataset with variable b-value configurations, whereas UNet was trained separately for each b-value configuration.

Fig. S4: top row: bar plot of the bias of the error of $D$ (left column), $D^{*}$ (middle column) and $f$ (right column) as a function of SNR for LSQ (orange), segmented fit (green), 1D NCDE (blue), UNet (pink) and Spatial NCDE (purple). Bottom row: bar plot of the mean of the variance per simulated parameter map of estimated IVIM parameters as a function of SNR for LSQ (orange), segmented fit (green), 1D NCDE (blue), UNet (pink) and Spatial NCDE (purple). Fig. S5: Top half parameter maps of the close-up views of the tumor region in patients with esophageal cancer. Parameters were estimated using LSQ, segmented fit, 1D NCDE, UNet and Spatial NCDE using all 13 available b-values. Overall, Spatial NCDE, UNet, 1D NCDE and segmented fit produced parameter maps with similar structural features and parameter values, while LSQ produced parameter maps which were dominated by the upper bound of the parameter range ($D^{*}$), or yielded spatially inconsistent attributions of signal decay to either $D$ or $f$ (white dotted ovals). While Spatial NCDE, UNet, 1D NCDE and segmented fit yielded more consistent parameter estimates some differences were apparent. 1D NCDE estimated higher $f$ values where $D^{*}$was close to 0, and thus their product harder to define (blue dotted ovals). Segmented fit yielded parameter maps for $D$ that were more smoothed compared to the estimations by LSQ, 1D NCDE, UNet and Spatial NCDE. Bottom half: parameter maps of the close-up views of the tumor region in patients with esophageal cancer estimated using only 9 of the available 13 b-values to determine the robustness to variations to the acquisition protocol. The UNet was not evaluated, since it required retraining to be evaluated on a different acquisition protocol. Overall, LSQ, segmented fit, 1D NCDE and Spatial NCDE produced parameter maps which were largely consistent with their respective estimations using 13 b-values (see top half). The most notable differences were that the estimated values for $D$ became substantially higher throughout the parameter map for the 1D NCDE, and that both the segmented fit and LSQ had a larger portion of estimates on the upper bound of the parameter range for $D^{*}$.

Fig. S6: parameter maps of the entire field-of-view in patients with esophageal cancer. Parameters were estimated using LSQ, segmented fit, 1D NCDE, UNet and Spatial NCDE.

Fig. S7. Top row: difference in sum of squared residuals (SSR) of the close-up views of the tumor region in patients with esophageal cancer between Spatial NCDE and LSQ, segmented fit, 1D NCDE and UNet, respectively. Red indicates an area where the reference method (either LSQ, segmented fit, 1D NCDE or UNet) has lower SSR than Spatial NCDE, while blue indicates an area where Spatial NCDE has lower SSR than the reference. Bottom three rows: difference in parameter estimates between Spatial NCDE and LSQ, segmented fit, 1D NCDE and UNet, respectively. Between Spatial NCDE and LSQ, LSQ has spatially inconsistent assignments of signal decay to either $D$ or $f$, while the Spatial NCDE-based estimations are more spatially coherent. Compared to the segmented fit, Spatial NCDE show a pattern where attributions to signal decay are assigned more to either $D$ or $f$, leading to difference maps for $D$ and $f$ which are roughly inverted. Compared to 1D NCDE, Spatial NCDEs parameter maps share the same structure but with a parameter-specific offset. Finally, the parameter maps for Spatial NCDE and UNet are very similar, especially for $D$ and $f$.

## S2.1 Methods

##### S2.1.1 Spatial NCDE architecture

| Layer Type | Output Shape | Number of model parameters |
| --- | --- | --- |
| $l_{\emptyset}^{1}$ | [batch size, 24, 24, 128] |  |
| └─ Linear | [batch size, 24, 24, 128] | 512 |
| └─ Rectified Linear Unit | [batch size, 24, 24, 128] | -- |
| └─ Linear | [batch size, 24, 24, 256] | 33,024 |
| └─ Rectified Linear Unit | [batch size, 24, 24, 256] | -- |
| └─ Linear | [batch size, 24, 24, 512] | 131,584 |
| └─ Rectified Linear Unit | [batch size, 24, 24, 512] | -- |
| └─ Linear | [batch size, 24, 24, 256] | 131,328 |
| └─ Rectified Linear Unit | [batch size, 24, 24, 256] | -- |
| └─ Linear | [batch size, 24, 24, 128] | 32,896 |
|  |  |  |
| $f_{\emptyset}$ |  |  |
| └─ Linear | [batch size, 24, 24, 128] | 16,512 (recursive) |
| └─ Rectified Linear Unit | [batch size, 24, 24, 128] | -- |
| └─ Linear | [batch size, 24, 24, 256] | 33,024 (recursive) |
| └─ Rectified Linear Unit | [batch size, 24, 24, 256] | -- |
| └─ Linear | [batch size, 24, 24, 512] | 131,584 (recursive) |
| └─ Rectified Linear Unit | [batch size, 24, 24, 512] | -- |
| └─ Linear | [batch size, 24, 24, 256] | 131,328 (recursive) |
| └─ Rectified Linear Unit | [batch size, 24, 24, 256] | -- |
| └─ Linear | [batch size, 24, 24, 384] | 98,688 (recursive) |
| └─ Tanh | [batch size, 24, 24, 384] | -- |
|  |  |  |
| $l_{\emptyset}^{2}$ |  |  |
| └─ Conv2D | [batch size, 24, 24, 64] | 73,792 |
| └─ Rectified Linear Unit | [batch size, 24, 24, 64] | -- |
| └─ Batch normalization | [batch size, 24, 24, 64] | 128 |
| └─ Conv2D | [batch size, 24, 24, 64] | 36,928 |
| └─ Batch normalization | [batch size, 24, 24, 64] | 128 |
| └─ Rectified Linear Unit | [batch size, 24, 24, 64] | -- |
| └─ MaxPool2D | [batch size, 12, 12, 64] | -- |
| └─ Conv2D | [batch size, 12, 12, 128] | 73,856 |
| └─ Rectified Linear Unit | [batch size, 12, 12, 128] | -- |
| └─ Batch normalization | [batch size, 12, 12, 128] | 256 |
| └─ Conv2D | [batch size, 12, 12, 128] | 147,584 |
| └─ Batch normalization | [batch size, 12, 12, 128] | 256 |
| └─ Rectified Linear Unit | [batch size, 12, 12, 128] | -- |
| └─ MaxPool2D | [batch size, 6, 6, 128] | -- |
| └─ Conv2D | [batch size, 6, 6, 256] | 295,168 |
| └─ Rectified Linear Unit | [batch size, 6, 6, 256] | -- |
| └─ Batch normalization | [batch size, 6, 6, 256] | 512 |
| └─ Conv2D | [batch size, 6, 6, 256] | 590,080 |
| └─ Batch normalization | [batch size, 6, 6, 256] | 512 |
| └─ Rectified Linear Unit | [batch size, 6, 6, 256] | -- |
| └─ ConvTranspose2D | [batch size, 12, 12, 128] | 131,200 |
| └─ Conv2D | [batch size, 12, 12, 128] | 295,040 |
| └─ Rectified Linear Unit | [batch size, 12, 12, 128] | -- |
| └─ Batch normalization | [batch size, 12, 12, 128] | 256 |
| └─ Conv2D | [batch size, 12, 12, 128] | 147,584 |
| └─ Batch normalization | [batch size, 12, 12, 128] | 256 |
| └─ Rectified Linear Unit | [batch size, 12, 12, 128] | -- |
| └─ ConvTranspose2D | [batch size, 24, 24, 64] | 32,832 |
| └─ Conv2D | [batch size, 24, 24, 64] | 73,792 |
| └─ Rectified Linear Unit | [batch size, 24, 24, 64] | -- |
| └─ Batch normalization | [batch size, 24, 24, 64] | 128 |
| └─ Conv2D | [batch size, 24, 24, 64] | 36,928 |
| └─ Batch normalization | [batch size, 24, 24, 64] | 128 |
| └─ Rectified Linear Unit | [batch size, 24, 24, 64] | -- |
| └─ ConvTranspose2D | [batch size, 24, 24, 4] | 2,308 |
|  |  |  |
| Total model parameters: |  | 2,680,132 |
| Trainable model parameters: |  | 2,680,132 |
| Non-trainable model parameters: |  | 0 |

Table S1: details of the architecture of the Spatial NCDE. A description of $l_{\emptyset}^{1}$, $f_{\emptyset}$ and $l_{\emptyset}^{2}$ can be found in section 2.2.1.

##### S2.1.2 Image acquisition

| Magnetic field strength (T) | 1.5 |
| --- | --- |
| Vendor / Type | Philips / Ingenia |
| Scan plane | Sagittal |
| Repetition time (ms) | 5967 |
| Echo time (ms) | 61 |
| Slice thickness (mm) | 4 |
| Flip angle (deg) | 90 |
| B-values (averages) | 0 (4), 10 (2), 20 (3), 30 (2), 40 (2), 50 (2), 75 (2), 100 (2), 200 (2), 350 (2), 500 (2), 650 (3), 800 |
| Fat suppression | SPIR |

Table S2: details of the acquisition protocol.

##### S2.1.3 Spatially coherent parameter maps

A base map ($M_{base}$) defined a global structure for all IVIM MRI parameters, which was generated based on the image size per dimension ($h$, w) and the Perlin noise scale per dimension ($S_{h},S_{w}$).This map was partitioned into $K$ distinct regions ($R_{k}$, where $k$ ∊ $[0, K-1]$), simulating different tissue types. Each region $R_{k}$ consisted of those voxels whose values were within the percentile range $[k\frac{100}{K},$ $(k+1)\frac{100}{K}]$ of $M_{base}$. The results for a map with $K=3$ regions are presented in Fig. S1.

Fig. S1: a) shared base map defining the global structure for all IVIM MRI parameters. b) the shared base map partitioned into three distinct, equally sized regions.

Each IVIM parameter was assigned a bounded range of values: $[0.0003 - 0.0035]$ $\frac{{mm}^{2}}{s}$ for $D$, $[0.005 - 0.1]$ $\frac{{mm}^{2}}{s}$ for $D^{*}$ and $[0 - 0.75]$for $f$. This range was divided into $n$ equally sized sub-ranges, where $n$ was a random draw from $[3, 4, 5]$. For each parameter, every region ($R_{k}$) was randomly assigned one of these sub-ranges, ensuring that each region had a unique combination of IVIM MRI parameter ranges, simulating distinct tissue types.

Within each region, voxel values for each parameter were generated using a new realization of Perlin noise. This approach ensured that parameter values within a region were both spatially dependent and non-uniform. For each parameter $p$, the values in region $R_{k}$ were computed as a weighted sum of two maps: an individual map specific to each parameter ($M_{p, unique}$) and a map shared among all parameters ($M_{p,shared}$):

$M_{p}=\lambda M_{p,unique}+(1-\lambda)M_{p,shared}$ , (6)

where $\lambda$ is a parameter-specific weighting value sampled from a uniform distribution with range [0, 0.5]. The value of $\lambda$ controls the degree of correlation between parameters: a higher $\lambda$ increases the influence of the shared map, resulting in greater inter-parameter correlation.

After computing $M_{p}$, the values in each region were normalized to the assigned sub-range of that parameter and region. This process resulted in a parameter map for each IVIM parameter, with distinct regions covering different sub-regions of the total parameter range. Examples of parameter maps sharing a spatial structure without consistent inter-parameter correlation are presented in Fig. S2.

Fig. S2: a) IVIM MRI parameter maps ($D$, $D^{*}$and $f$, respectively) showing shared spatial structure. b) 2D distribution plots showing no consistent inter-parameter correlation based on parameter values.

##### S2.1.4 In-vivo preprocessing

Diffusion-weighted images were denosied using Patch2Self, a self-supervised denoising approach [30,31]. All signal curves were normalized with respect to their signal at $b=0$.

Effective in-vivo training of the 1D NCDE, UNet and Spatial NCDE required careful data curation to exclude uninformative or unrepresentative voxels (e.g., background or out-of-bound signal curves). Including such voxels can hinder convergence by introducing infeasible training targets. To address this, we generated a voxel mask by computing the upper and lower signal bounds based on the bounds of the parameter space (e.g. the upper signal bound is realized by computing signals from the lower bound of $D$, lower bound of $D^{*}$ and lower bound of $f$). Voxels were included in the mask if more than 50% of their measurements across b-values lie within these bounds.

For voxel-wise models, training samples were drawn exclusively from masked voxels. For spatially aware models, input patches were drawn containing both masked and unmasked voxels while the loss was computed only over the masked subset.

## S2.2 Results

Fig. S3: Box plots of the distribution of MSEs over 1000 simulated parameter maps for $D$ (top row), $D^{*}$ (middle row) and $f$ (bottom row) as a function of SNR (columns from left to right: SNR 5, 10, 20, 30, 40) and as a function of sequence length (x-axes) for LSQ (orange), segmented fit (green), 1D NCDE (blue), UNet (pink) and Spatial NCDE (purple). B-values used for evaluation can be found in Table 1. The segmented fit could not be performed on 5 b-values, as the algorithm required more b-values for the multi-step approach. The white circle represents the MSE over simulated parameter maps. Across parameters and SNR levels, the Spatial NCDE consistently showed lower MSEs compared to all other estimation methods. Note that the NCDE and Spatial NCDE were trained once on a dataset with variable b-value configurations, whereas UNet was trained separately for each b-value configuration.

Fig. S4: top row: bar plot of the bias of the error of $D$ (left column), $D^{*}$ (middle column) and $f$ (right column) as a function of SNR for LSQ (orange), segmented fit (green), 1D NCDE (blue), UNet (pink) and Spatial NCDE (purple). Bottom row: bar plot of the mean of the variance per simulated parameter map of estimated IVIM parameters as a function of SNR for LSQ (orange), segmented fit (green), 1D NCDE (blue), UNet (pink) and Spatial NCDE (purple).

|  | parameter | Effect size (p-value) | | | |
| --- | --- | --- | --- | --- | --- |
|  |  | LSQ | Segmented fit | 1D NCDE | UNet |
| SNR5 | $D$ | *73% (< 0.05)* | *75% (< 0.05)* | *14% (< 0.05)* | *29% (< 0.05)* |
|  | $f$ | *70% (< 0.05)* | *85% (< 0.05)* | *34% (< 0.05)* | *41% (< 0.05)* |
|  | $D^{*}$ | *58% (< 0.05)* | *58% (< 0.05)* | *-10% (< 0.05)* | *6% (< 0.05)* |
| SNR10 | $D$ | *84% (< 0.05)* | *87% (< 0.05)* | *59% (< 0.05)* | *49% (< 0.05)* |
|  | $f$ | *87% (< 0.05)* | *91% (< 0.05)* | *58% (< 0.05)* | *48% (< 0.05)* |
|  | $D^{*}$ | *86% (< 0.05)* | *89% (< 0.05)* | *71% (< 0.05)* | *64% (< 0.05)* |
| SNR20 | $D$ | *81% (< 0.05)* | *85% (< 0.05)* | *62% (< 0.05)* | *55% (< 0.05)* |
|  | $f$ | *83% (< 0.05)* | *88% (< 0.05)* | *52% (< 0.05)* | *44% (< 0.05)* |
|  | $D^{*}$ | *85% (< 0.05)* | *89% (< 0.05)* | *71% (< 0.05)* | *67% (< 0.05)* |
| SNR30 | $D$ | *545 (< 0.05)* | *66% (< 0.05)* | *41% (< 0.05)* | *64% (< 0.05)* |
|  | $f$ | *60% (< 0.05)* | *70% (< 0.05)* | *17% (< 0.05)* | *49% (< 0.05)* |
|  | $D^{*}$ | *72% (< 0.05)* | *83% (< 0.05)* | *53% (< 0.05)* | *68% (< 0.05)* |
| SNR40 | $D$ | *55% (< 0.05)* | *67% (< 0.05)* | *53% (< 0.05)* | *58% (< 0.05)* |
|  | $f$ | *60% (< 0.05)* | *71% (< 0.05)* | *32% (< 0.05)* | *42% (< 0.05)* |
|  | $D^{*}$ | *80% (< 0.05)* | *88% (< 0.05)* | *70% (< 0.05)* | *75% (< 0.05)* |

*Table S3: Overview of effect sizes and p-values of the MSE in simulated data for Spatial NCDE compared to LSQ, segmented fit, 1D NCDE and UNet. Spatial NCDE outperformed all other estimation methods by estimating lower MSEs for 14/15 comparisons across parameters and SNR levels (except for 1D NCDE estimating lower MSE for* $D^{*}$ *at SNR5). All differences were significant (p-value < 0.05). The effect size was computed as* $\frac{{MSE}_{reference}-{MSE}_{SpatialNCDE}}{{MSE}_{reference}}x100\%$*.*

|  | Inference speed | Training time per iteration |
| --- | --- | --- |
| LSQ | 2000 voxels in 0.31 s | Not applicable |
| Segmented fit | 2000 voxels in 0.42 s | Not applicable |
| 1D NCDE | 50176 voxels in 2.05 s | 9.07 s |
| UNet | 50176 voxels in 0.003 s | 2.22 s |
| Spatial NCDE | 50176 voxels in 2.1 s | 41.03 s |

Table S4: Overview of inference speed and training time per iteration for LSQ, segmented fit, 1D NCDE, UNet and Spatial NCDE. While the Spatial NCDE training was substantially slower than UNet, the inference speed was comparable to that of the 1D NCDE and substantially faster than LSQ and segmented fit. Spatial NCDE predicted a 224 x 224 slice in 2.05 seconds on an NVIDIA A100 GPU, reaching clinically feasible speeds.

Fig. S5: Top half parameter maps of the close-up views of the tumor region in patients with esophageal cancer. Parameters were estimated using LSQ, segmented fit, 1D NCDE, UNet and Spatial NCDE using all 13 available b-values. Overall, Spatial NCDE, UNet, 1D NCDE and segmented fit produced parameter maps with similar structural features and parameter values, while LSQ produced parameter maps which were dominated by the upper bound of the parameter range ($D^{*}$), or yielded spatially inconsistent attributions of signal decay to either $D$ or $f$ (white dotted ovals). While Spatial NCDE, UNet, 1D NCDE and segmented fit yielded more consistent parameter estimates some differences were apparent. 1D NCDE estimated higher $f$ values where $D^{*}$was close to 0, and thus their product harder to define (blue dotted ovals). Segmented fit yielded parameter maps for $D$ that were more smoothed compared to the estimations by LSQ, 1D NCDE, UNet and Spatial NCDE. Bottom half: parameter maps of the close-up views of the tumor region in patients with esophageal cancer estimated using only 9 of the available 13 b-values to determine the robustness to variations to the acquisition protocol. The UNet was not evaluated, since it required retraining to be evaluated on a different acquisition protocol. LSQ, Overall, segmented fit, 1D NCDE and Spatial NCDE produced parameter maps which were largely consistent with their respective estimations using 13 b-values (see top half). The most notable differences were that the estimated values for $D$ became substantially higher throughout the parameter map for the 1D NCDE, and that both the segmented fit and LSQ had a larger portion of estimates on the upper bound of the parameter range for $D^{*}$.

Fig. S6: parameter maps of the entire field-of-view in patients with esophageal cancer. Parameters were estimated using LSQ, segmented fit, 1D NCDE, UNet and Spatial NCDE.

Fig. S7. Top row: difference in sum of squared residuals (SSR) of the close-up views of the tumor region in patients with esophageal cancer between Spatial NCDE and LSQ, segmented fit, 1D NCDE and UNet, respectively. Red indicates an area where the reference method (either LSQ, segmented fit, 1D NCDE or UNet) has lower SSR than Spatial NCDE, while blue indicates an area where Spatial NCDE has lower SSR than the reference. Bottom three rows: difference in parameter estimates between Spatial NCDE and LSQ, segmented fit, 1D NCDE and UNet, respectively. Between Spatial NCDE and LSQ, LSQ has spatially inconsistent assignments of signal decay to either $D$ or $f$, while the Spatial NCDE-based estimations are more spatially coherent. Compared to the segmented fit, Spatial NCDE show a pattern where attributions to signal decay are assigned more to either $D$ or $f$, leading to difference maps for $D$ and $f$ which are roughly inversted. Compared to 1D NCDE, Spatial NCDEs parameter maps share the same structure but with a parameter-specific offset. Finally, the parameter maps for Spatial NCDE and UNet are very similar, especially for $D$ and $f$.
